# Transcriptional Differences in Peanut (*Arachis hypogaea* L.) Seeds at the Freshly Harvested, After-ripening and Newly Germinated Seed Stages: Insights into the Regulatory Networks of Seed Dormancy Release and Germination

**DOI:** 10.1371/journal.pone.0219413

**Published:** 2020-01-03

**Authors:** Pingli Xu, Guiying Tang, Weipei Cui, Guangxia Chen, Chang-Le Ma, Jieqiong Zhu, Pengxiang Li, Lei Shan, Zhanji Liu, Shubo Wan

**Affiliations:** 1 Bio-Tech Research Center, Shandong Academy of Agricultural Sciences / Shandong Provincial Key Laboratory of Crop Genetic Improvement, Ecology and Physiology, Jinan, Shandong, China; 2 College of Life Science, Shandong Normal University, Jinan, Shandong, China; 3 Shandong Academy of Grape, Jinan, Shandong, China; 4 Shandong Cotton Research Center, Shandong Academy of Agricultural Sciences, Jinan, Shandong, China; Huazhong University of Science and Technology, CHINA

## Abstract

Seed dormancy and germination are the two important traits related to plant survival, reproduction and crop yield. To understand the regulatory mechanisms of these traits, it is crucial to clarify which genes or pathways participate in the regulation of these processes. However, little information is available on seed dormancy and germination in peanut. In this study, seeds of the variety Luhua No.14, which undergoes nondeep dormancy, were selected, and their transcriptional changes at three different developmental stages, the freshly harvested seed (FS), the after-ripening seed (DS) and the newly germinated seed (GS) stages, were investigated by comparative transcriptomic analysis. The results showed that genes with increased transcription in the DS vs FS comparison were overrepresented for oxidative phosphorylation, the glycolysis pathway and the tricarboxylic acid (TCA) cycle, suggesting that after a period of dry storage, the intermediates stored in the dry seeds were rapidly mobilized by glycolysis, the TCA cycle, the glyoxylate cycle, etc.; the electron transport chain accompanied by respiration was reactivated to provide ATP for the mobilization of other reserves and for seed germination. In the GS vs DS pairwise comparison, dozens of the upregulated genes were related to plant hormone biosynthesis and signal transduction, including the majority of components involved in the auxin signal pathway, brassinosteroid biosynthesis and signal transduction as well as some GA and ABA signal transduction genes. During seed germination, the expression of some *EXPANSIN* and *XYLOGLUCAN ENDOTRANSGLYCOSYLASE* genes was also significantly enhanced. To investigate the effects of different hormones during seed germination, the contents and differential distribution of ABA, GAs, BRs and IAA in the cotyledons, hypocotyls and radicles, and plumules of three seed sections at different developmental stages were also investigated. Combined with previous data in other species, it was suggested that the coordination of multiple hormone signal transduction nets plays a key role in radicle protrusion and seed germination.

## Introduction

Seed dormancy and germination are the two important traits in the plant life cycle involved in species survival and offspring proliferation. Different plant species present various classes of dormancy to regulate the timing of seed germination and to help seedlings emerge under favorable conditions. Primary dormancy of seeds is acquired during the seed maturation phase and reaches a high level in freshly harvested seeds. During the subsequent dry period of seeds (so-called after-ripening, AR), primary dormancy slowly decreases. When the dormancy level gradually decreases, seeds can rapidly exit dormancy and proceed to germination during imbibition under favorable conditions [1]. A recent study in *Arabidopsis* suggested that seed AR is a specific developmental pathway that is independent of germination potential and does not rely on ABA regulation [2]. The dormancy alleviation in dry seeds is associated with the production of ROS and the carbonylation of specific embryo proteins [3–5]. Concomitantly, the metabolic switches between different developmental periods of seeds are also relevant to the distinct expression profiles of genes involved in several metabolic pathways [6].

Strictly defined, germination is the initial emergence of the radicle from the seed coat. In some species such as *Arabidopsis*, whose embryo is enclosed by the endosperm and the surrounding testa, seed germination consists of two visible steps: first, the testa ruptures due to expansion of the endosperm and embryo, followed by radicle protrusion through the endosperm. However, in leguminous plants, seeds have no endosperm, and testa splitting marks the completion of germination [7–9]. Phytohormones play important roles in the induction and maintenance of seed dormancy as well as the release of dormancy and subsequent germination. Abscisic acid (ABA) and gibberellins (GAs) negatively and positively regulate seed germination, respectively. In different development stages of the seed, the ratio of ABA to GA in embryos is dynamic. Dormant seeds maintain a high ABA/GA ratio, and dormancy maintenance also depends on a high ABA/GA ratio, while dormancy release involves a net shift to increased GA biosynthesis and ABA degradation, resulting in a low ABA/GA ratio and seed germination associated with increased GA content and sensitivity [7, 10]. The basic role of auxin is to promote cell elongation. An increased GA content leads to an obvious change in auxin content and transport during seed germination. A peak in free IAA occurs prior to the initiation of radicle elongation [11]. Brassinosteroids (BRs), which constitute another antagonist of ABA, and GAs play parallel roles in promoting cell elongation and germination. Photodormancy is released by the GA/light signal transduction pathway, while subsequent endosperm rupture is activated by the BR and GA/light pathways via distinctly different mechanisms [11–13].

In recent years, many studies have focused on gene expression analyses related to seed dormancy and germination and have revealed genes that regulate these processes, especially genes involved in phytohormone signaling, such as those involved in the ABA, GA, BR and IAA pathways [9, 14–22]. Many transcriptome analyses involved in seed dormancy and germination in different plant species have described a global view of gene expression changes among different developmental stages, in different regions of seeds, or in dormant and nondormant seeds [15, 23–25]. Bassel et al. (2011) indicated that the characteristics of seed dormancy and germination are highly conserved in the evolution of flowering plants. A genome-wide transcriptional analysis of dormant and after-ripened *Arabidopsis* seeds at four time points and in two seed compartments showed that gene sets strongly enhanced at the initiation of imbibition are overrepresented for GO (Gene Ontology) classes including key cellular metabolic processes such as translation and amino acid, organic acid, nucleotide and carbohydrate metabolism, and the downregulated sets included classes associated with response to stress and other environmental cues [15]. During the germination of soybean seeds, the GA, ethylene and BR pathways are transcriptionally active, while ABA signaling is downregulated in the embryonic axes [26].

Cultivated peanut (*Arachis hypogaea* L.) is a distinct oilseed crop species that flowers aboveground and fructifies belowground. After peanut maturity, delayed harvest may cause seeds vivipary under conditions of continuous rainy days, which always leads to a reduction in yield and seed quality. The species *A*. *hypogaea* L. is divided into two subspecies: *A*. *hypogaea* subsp. *hypogaea* and *A*. *hypogaea* subsp. *fastigiata*. In the subspecies *A*. *hypogaea* subsp. *hypogaea* var. *hypogaea* (Virginia and Runner market types) and var. *hursita*, varieties have longer growth cycles, and seeds have longer dormancy stages. However, in subspecies *A*. *hypogaea* subsp. *fastigiata* var. *fastigiata* (Valencia market class) and var. *vulgaris* (Spanish market type), varieties mature early but generally lack fresh seed dormancy [27]. However, even the subsp. *hypogaea* (which undergoes dormancy), the dormancy status is easily broken during storage for a short time at room temperature. In our present research, to explore the regulatory mechanisms of dormancy release and germination of peanut seeds, the seeds of the variety Luhua No.14 (LH14), which belongs to the subspecies *hypogaea* and whose seeds undergo nondeep dormancy, were selected, and their transcriptional changes at three developmental stages, which are the freshly harvested (FS), after-ripening (DS) and newly germinated seed (GS) stages, were investigated by comparative transcriptomic analysis.

## Materials and methods

### Plant material and growth conditions

The peanut variety LH14 was bred by Shandong Peanut Research Institute and was planted by our group in the field at Yinmaquan Farm for subsequent assays and analyses. The seeds were harvested from the field and divided into two portions. One portion of the freshly harvested seeds was kept in paper bags under -80°C or under ambient temperature and humidity, and another portion designated as after-ripening seeds was dried in the sun for more than two weeks and then kept in paper bags at room temperature. For the assay of germination rate, 5 accessions [Chico (CHI) and Silihong (SLH) from subspecies *fastigiata*, and LH14, Fenghua No.1 (FH1) and Linguimake (LGMK) from subspecies *hypogaea*] were selected. Thirty-six seeds from each accession were sown in three petri dishes with four layers of absorbent gauze wetted with demineralized water and incubated in a 15°C incubator in darkness, and germination assay was repeated for three times. The status of imbibition was determined at 24 h intervals based on the changes in seed swelling, and the seeds were considered germinated when the radicles broke through the seed coat.

### RNA extraction, library construction and sequencing

At each of the FS, DS, and GS stages, whole seeds were collected for RNA extraction. Two biological replicates were established, which has potentially interfered with statistical analysis of gene expression. Total RNA of the samples was isolated using the improved CTAB method [28] and was treated with DNase I (RNase-free) according to the TaKaRa’s protocol. The quantity and purity of the samples were measured using a Qubit^®^ RNA Assay Kit by a Qubit^®^ 2.0 Fluorometer (Life Technologies, CA, USA) and a NanoPhotometer^®^ spectrophotometer (IMPLEN, CA, USA), and the integrity was examined with a RNA Nano 6000 Assay Kit of the Bioanalyzer 2100 system (Agilent Technologies, CA, USA).

A total of 3 μg of RNA from each sample was used to construct a cDNA library. The libraries were generated using a NEBNext^®^ Ultra^™^ RNA Library Prep Kit for Illumina^®^ (New England Biolabs, Ipswich, MA, USA), following the manufacturer’s recommendations. Sequencing of the six libraries was completed by Beijing Novogene Bioinformatics Technology Co., Ltd., on the HiSeq 2000 platform (Illumina, San Diego, CA, USA), and 100 bp paired-end reads were generated.

### Data analysis for RNA-Seq

The raw data in fastq format were cleaned by removing adapter sequences, reads containing poly-N sequences, and low-quality reads (Q≤20). The clean reads were then aligned to the reference genome (*A*. *duranensis*, https://www.peanutbase.org/home) using the TopHat v2.0.12. All sequence data (Bioproject_accession: PRJNA545858) were submitted to the BioProject database of the National Center for Biotechnology Information (NCBI). The expression levels were calculated with Cufflinks and normalized by the fragments per kilobase of transcript per million mapped reads (FPKM) method [29].

The differentially expressed genes (DEGs) between two samples were detected by the DESeq R package (ver. 1. 18. 0), and the *P* value was adjusted using the Benjamini and Hochberg method to control the false discovery rate [30]. Genes with an adjusted *P<*0.05 were considered as the DEGs. Annotation of gene function was performed by comparisons with the information in the nonredundant nucleotide and protein sequences (NCBI) and the protein sequence database Swiss-Prot. GO and KEGG (Kyoto Encyclopedia of Genes and Genomes) enrichment analyses were performed to identify which DEGs were significantly enriched in GO terms or metabolic pathways by the GOseq R package and KOBAS software. GO terms with corrected *P* values less than 0.05 were considered significantly enriched by DEGs. The GO annotations were functionally classified by the WEGO (Web Gene Ontology Annotation Plot) software for gene function distribution. Those genes with at least twice the level of expression at specific developmental stages were defined as preferential DEGs in KEGG pathways in reference to soybean.

### Real-time quantitative RT-PCR (qRT-PCR) analysis

DEGs preferentially expressed in specific metabolic pathways and hormone signal pathways were selected for validation using qRT-PCR. The total RNAs of the FS, DS and germinated seeds (firstly, seeds imbibited for 4h, then germinated for 48h) were isolated using the improved CTAB method [28], and then, their first-strand cDNAs were synthesized according to the instruction of PrimeScript RT reagent kit with gDNA eraser (TaKaRa Biotechnology, Dalian, China). The candidate genes and their amplification primers used are listed in Table 1. qRT-PCR was performed using SYBR Premix Ex Taq (TliRNaseH Plus; TaKaRa Biotechnology, Dalian, China). The reaction conditions were as follows: predenaturing for 5 min at 94°C, followed by 40 cycles of 15 s at 94°C and 30 s at 60°C. The relative expression levels of the target genes were analyzed in conjunction with *AhACTIN7* serving as an internal control and were calculated using the 2^-ΔΔCt^ method [31].

**Table 1:** Primers used for verification of gene expression levels by qRT-PCR.

Gene ID	Forward Primer	Reverse Primer	Annotation/Abbreviation	Pathway
Aradu.7JD6C	CCTGATGTAGTGGGATCATTCG	GCCACTGGAGCCATTCTAAA	NADH-ubiquinone oxidoreductase chain 1/MT-ND1	OP/complex I
Aradu.FMP57	CGACGAGTACACTAAGGAGAGA	TGGGCTGAAGTGACTTGATATG	Succinate dehydrogenase [ubiquinone] iron-sulfur subunit 2/SDH2-2	OP/complex II
Aradu.77KSP	CGATTCGTCGTGGTCATCAA	GCCGTCAACTACCTCAATCTC	Cytochrome c1-1, heme protein/CYCL	OP/complex III
Aradu.UQR72	CCTTTCCATGAAGCACGAGTA	AGGAATGGTGCGTTGATATAGG	Cytochrome b-c1 complex subunit 7-1/QCR7-1	OP/complex III
Aradu.BDB6C	CCACGTTGGAAGGACATCATA	GCCAAATACCTCCGATCTCTAC	Cytochrome c oxidase subunit 3/COX3	OP/complex IV
Aradu.4B6K6	GGTGACTCTCCCAGTGTTAATG	CCTTAGCCTTCTCACCCAATATG	ATP synthase subunit gamma/ATPC	OP/complex V
Aradu.71QRQ	CTACCTGCCATCTCTGCATTTA	CCAACCTCTTCACCCATCAA	Pyruvate dehydrogenase E1 component subunit beta-1/PDH-E1	GG
Aradu.HH99D	CCGAGGTCACAGGAAGTAATG	GCTGGTATCCAGGCAAGAAA	Formate dehydrogenase/ FDH	TCA
Aradu.8XX6M	CTGTCATGGGCCAGAATCTT	CTCGTTCAACGGTCTCATCTAC	6-Phosphogluconate dehydrogenase, decarboxylating 3/6-PGDH	GG
Aradu.H3G7C	CTCCTTCTCTTCTCCCATCAATC	CTTCCATATAACCCTCGTCATCTC	Isocitrate dehydrogenase [NADP]/IDH	TCA
Aradu.RB3TY	CCAAGTGGAGTTCAGCTAAGAG	CGAAATCTGCAGCTCTGTCTAT	2-oxoglutarate dehydrogenase/OGDH	TCA
Aradu.52T5J	CTTGTCTCAAGGGCCTCAAT	TTCCTCCACACCGTTCTTTC	Malate dehydrogenase/MDH	TCA
Aradu.U0HC6	AGAAGAGTAAGAGCAGCGAAAC	CAATCGCTATCCCGTCCATATT	Auxin-responsive protein IAA13/IAA13	AuS
Aradu.06HW9	CCTCTTCTCACTCTCCACTCT	GCTCTGCAAGACAACGATTTG	Auxin efflux carrier component 2/PIN2	AuS
Aradu.PZ2UH	GGGAAAGCTCAGGAAGGAAA	CTCTCGGCTCTGATCTTGAATC	Auxin response factor 3/ARF3	AuS
Aradu.MF3XQ	AGGCAGGATCTGTAGGAAGA	GTGTCATTCAGCAGACCATCTA	Auxin response factor 5/ARF5	AuS
Aradu.FVI2X	CCTTAGATGGGTCATGGAGAAA	GAAGAGTGGGTGGTTGAAGTA	Auxin-responsive protein SAUR32/SAUR32	AuS
Aradu.DSS3T	AAAGTGGGTGGATGGCTATG	GTAGTGACCCTGAATGACAAAGA	Expansin-A11/EXPA11	-
Aradu.V7R7T	CTGGAACTATGGGAGGTTCTTG	AGCTGAAGCCATCGTTGTATAG	Expansin-A3/EXPA3	-
Aradu.R6HZB	TAGCACCAGAGCCTTTCAATC	CTTGATTCTTCAACGCGAACAG	Expansin-like A1/EXLA1	-
Aradu.OQC7R	GGATTCACGCAAACAATGGAG	GTCACTTGGAACCTCAAGAAGA	Expansin-like B1/EXLB1	-
Aradu.4I7WA	CCACCAAGAACTTCCACACTTA	GGGAAAGGAACACCCATAGATT	Xyloglucan endo-transglucosylase / hydrolase 2 / XTHL2	-
Aradu.AA5UH	CAACAGCCTATGGAACGCAG	CGGAGGTTTCACAGCCATCA	Probable xyloglucan endo-transglucosylase / hydrolase protein B / XTHLB	-

OP: oxidative phosphorylation; GG: glycolysis; TCA: tricarboxylic acid cycle; AuS: Auxin signaling

### Hormone extraction and quantification

One gram of samples was ground with a mortar and pestle in liquid nitrogen and extracted in cold 80% (v/v) methanol with butylated hydroxytoluene (1 mmol·L^-1^) overnight at 4°C. The extracts were collected after centrifugation at 10000 g for 20 min at 4°C, purified through a C_18_ Sep-Park Cartridge (Waters Crop., Milford, MA) and then dried under N_2_. The hormone fractions were dissolved in phosphate-buffered saline (PBS; 0.01 mol·L^-1^, pH 7.4) with 0.1% (v/v) Tween-20 and 0.1% (w/v) gelatin for determining the levels of hormones by ELISA (Enzyme-Linked Immunosorbnent Assay).

The peanut seeds from the freshly harvested, and imbibition for 4h, 16h, 28h and 52h were dissected into three parts, respectively: cotyledons (COs), hypocotyls and radicles (HRs), and the remaining plumules (PLs). The contents of ABA, GA_3_, IAA and BRs in these three parts at the five stages mentioned above were detected by ELISA according to the method reported by Yang et al. [32]. Monoclonal antibodies against ABA, GA_3_, IAA and BRs produced by the Phytohormones Research Institute, China Agricultural University, were used as the first antibodies, and IgG (Immuno globulin G) horseradish peroxidase was used as the secondary antibody. The content of each hormone was calculated via known amounts of standard hormones added to the extracts, according to the methods of Weiler et al. [33].

### Statistical analyses

One-way analyses of variance (ANOVA) were used to analyze the effects between two different status (fresh or dry seeds) of one variety on germination ratio, and two-ways ANOVA were applied to check out the effects among different varieties on germination ratio. Mean differences were determined based on the least significant difference (LSD) at the P<0.05 probability level, and shown alphabetically in order a, b, and so on. Mean differences of the gene expression levels and phytohormone contents among different stages of seeds were evaluated by one-way ANOVA, and the significant differences at 5% level were shown as different letters. The SPSS statistical software was used for all analyses.

## Results

### Germination assays

**Fig 1:**
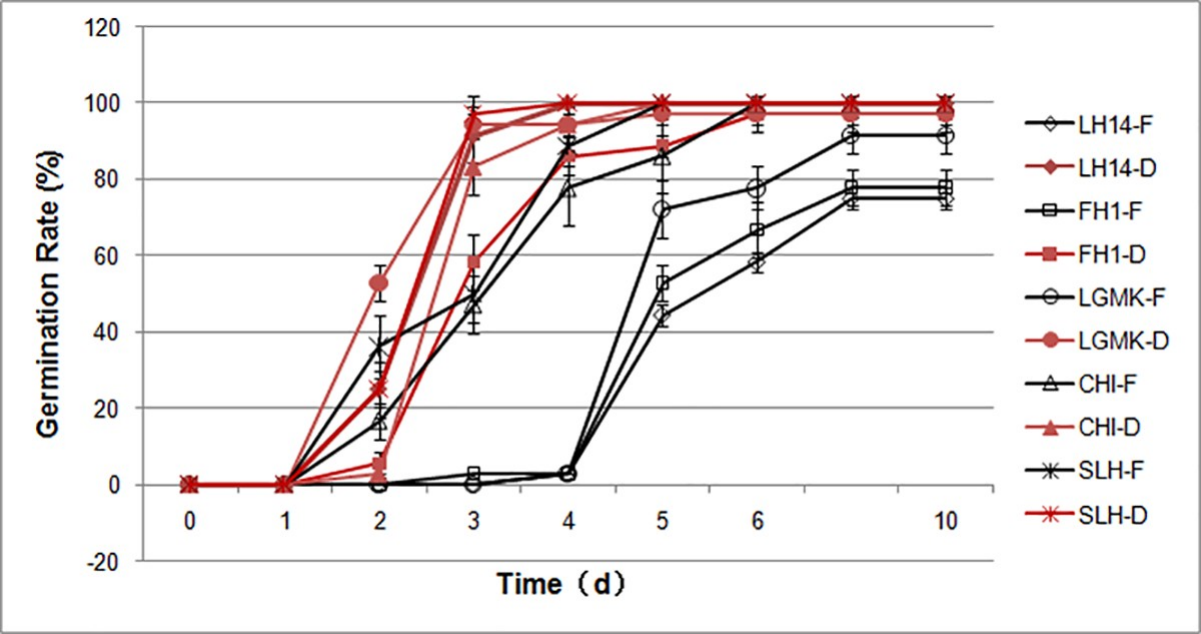
Germination rate of freshly harvested (F) peanut seeds and of seeds after drying (D) from different varieties. LH14: Luhua No. 14, FH1: Fenghua No. 1, LGMK: Linguimake, CHI: Chico, SLH: Silihong.

To explore the influence of dry storage on seed germination, germination assays of freshly harvested seeds and dry seeds stored for more than two weeks were performed. It was found that in the CHI and SLH nondormant peanut seeds, there were no obvious differences in the beginning time of germination between the freshly harvested seeds and the dry seeds; these seeds began to germinate on the second day, but the uniformity of the germination of the dry seeds was much higher than that of the fresh seeds (Fig 1). However, the freshly harvested seeds of LH14, FH1 and LGMK with nondeep dormancy began to germinate at four days after imbibition (DAI), but their germination rates reached only 75%, 77.8%, and 91.7% at 10 DAI. After a period of dry storage, the majority of dried seeds started to germinate at two DAI, and their germination rates significantly increased at four DAI and reached 100%, 97.2%, and 97.2% at germination six DAI (Fig 1, S1 Table). As well as, there were no significant differences in germination rates between the nondormant seeds and the dried seeds with nondeep dormancy (Fig 1, S1 Table). It was suggested that the dormancy of the nondeep dormant peanut seeds was released by the process of after-ripening during dry storage, whereas the nondormant peanut seeds did not undergo the typical after-ripening procedure. Thus, the peanut variety LH14 with nondeep dormancy was selected for subsequent experiments.

### Transcriptome sequencing and assessment

To investigate the changes in the transcript profiles among LH14 seeds at FS, DS, and GS stages, RNA-seq of six samples from the three stages was performed by Illumina sequencing. In total, 44.5 to 63.4 million raw reads from the six libraries (with an error rate of approximately 0.03%) were generated, and 44.1~62.7 million clean reads generated by removing low-quality sequences were selected for further analysis (Table 2). Among these reads, 81.11~85% were mapped to the reference genome (*A*. *duranensis*, https://www.peanutbase.org/home), and the reads uniquely mapped accounted for 78.34~82.63%. The percentages of reads mapped to exon, intron, and intergenic regions were 83.1~87.5%, 1.3~3.6%, and 11.1~15.1%, respectively.

**Table 2:** Summary of the transcriptome sequencing data.

Sample_name	FS_1	FS_2	DS_1	DS_2	GS_1	GS_2
Raw reads	59931664	44502832	56994704	63409368	50687486	51665824
Clean reads	59307780	44076312	56410796	62736078	50126196	50974284
Q30 (%)	93.56	93.72	93.70	93.46	93.18	91.92
Total mapped	48105991 (81.11%)	36147297 (82.01%)	45822510 (81.23%)	50745769 (80.89%)	42171822 (84.13%)	43329793 (85%)
Uniquely mapped	46684962 (78.72%)	35267221 (80.01%)	44419221 (78.74%)	49145234 (78.34%)	40939147 (81.67%)	42120239 (82.63%)
Exon	83.7%	87.5%	85.5%	84.5%	83.1%	83.1%
Intron	1.3%	1.4%	1.8%	2.6%	3.1%	3.6%
Intergenic	15.1%	11.1%	12.7%	12.8%	13.9%	13.2%

### Analysis of DEGs at different stages

**Fig 2:**
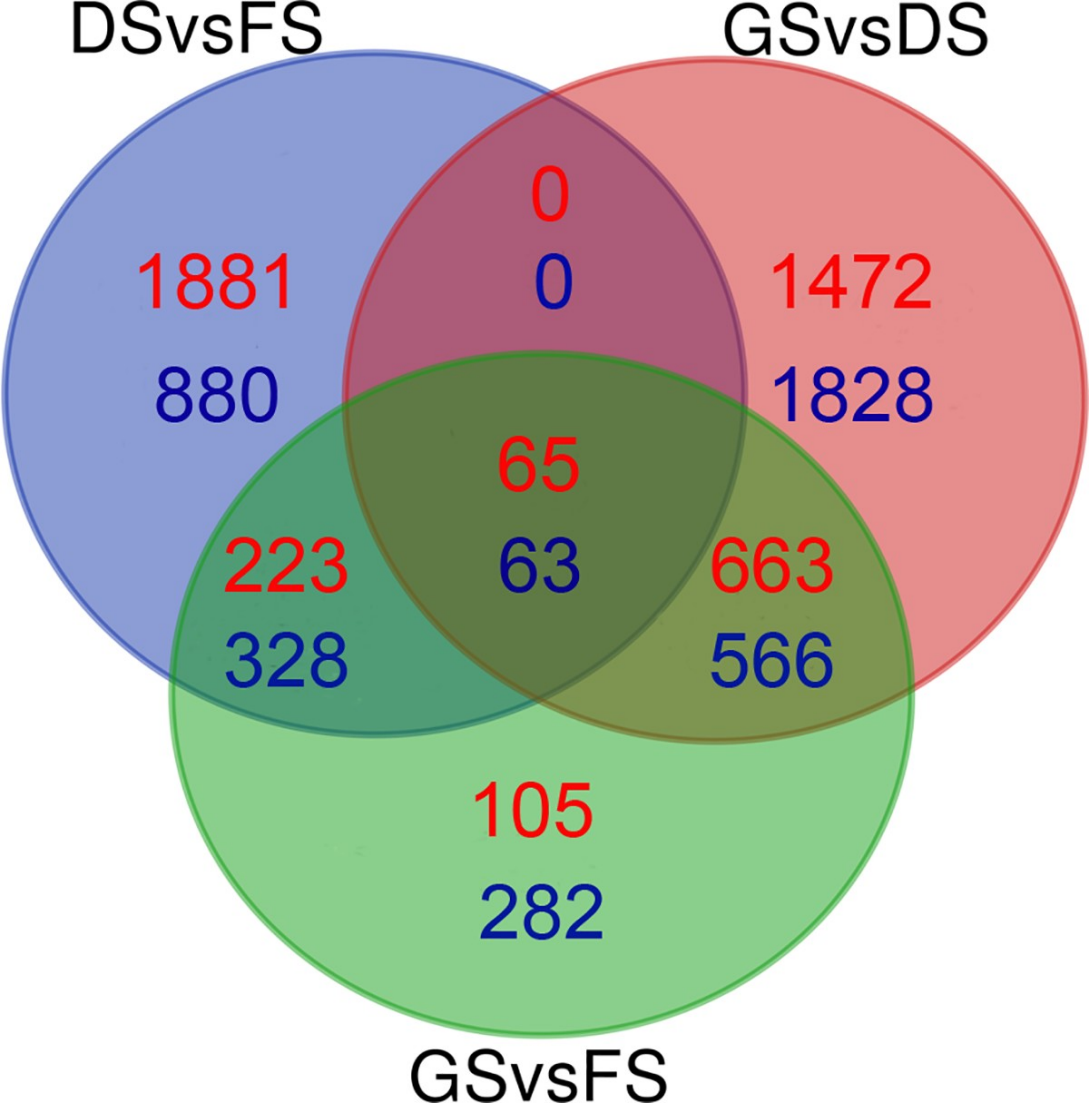
Venn diagrams of differentially expressed genes (DEGs) among seeds during the freshly harvested (FS), after-ripening (DS), and newly germinated (GS) seed stages. The red counts represent upregulation, and the blue counts represent downregulation between the different comparisons.

To investigate the major genes controlling the dormancy release and germination of peanut seeds, analyses of the DEGs in the three sample pairs (DS vs FS, GS vs FS, and GS vs DS) were performed; totals of 3440, 2295, and 4657 DEGs were identified in the above comparison pairs, respectively. There were 2169 upregulated and 1271 downregulated genes between the after-ripening and the freshly harvested seeds, and there were 1056 upregulated and 1239 downregulated genes between the newly germinated and the freshly harvested seeds. Between the newly germinated and the AR stages, there were many more DEGs than those in the other two comparisons; among them, the expression level of 2200 genes increased, and that of 2457 genes decreased. Of the total 5425 upregulated and 4967 downregulated DEGs, only 65 and 63 DEGs were common across all three stages, respectively, and approximately 288, 728 and 65 upregulated DEGs and 391, 629 and 63 downregulated DEGs overlapped in the different respective combinations of sample pairs; moreover, 1881 and 880, 282 and 105, and 1472 and1828 upregulated and downregulated genes were specifically found in the DS vs FS, GS vs FS, and GS vs DS comparisons, respectively (Fig 2). Interestingly, although the morphological differences, such as the size of cotyledons, the length of radicles, and so on, between the newly germinated seeds and the freshly harvested seeds were the most obvious, this comparison showed fewer DEGs than either of the other pairwise comparisons.

### GO functional classification of DEGs at different stages

**Fig 3:**
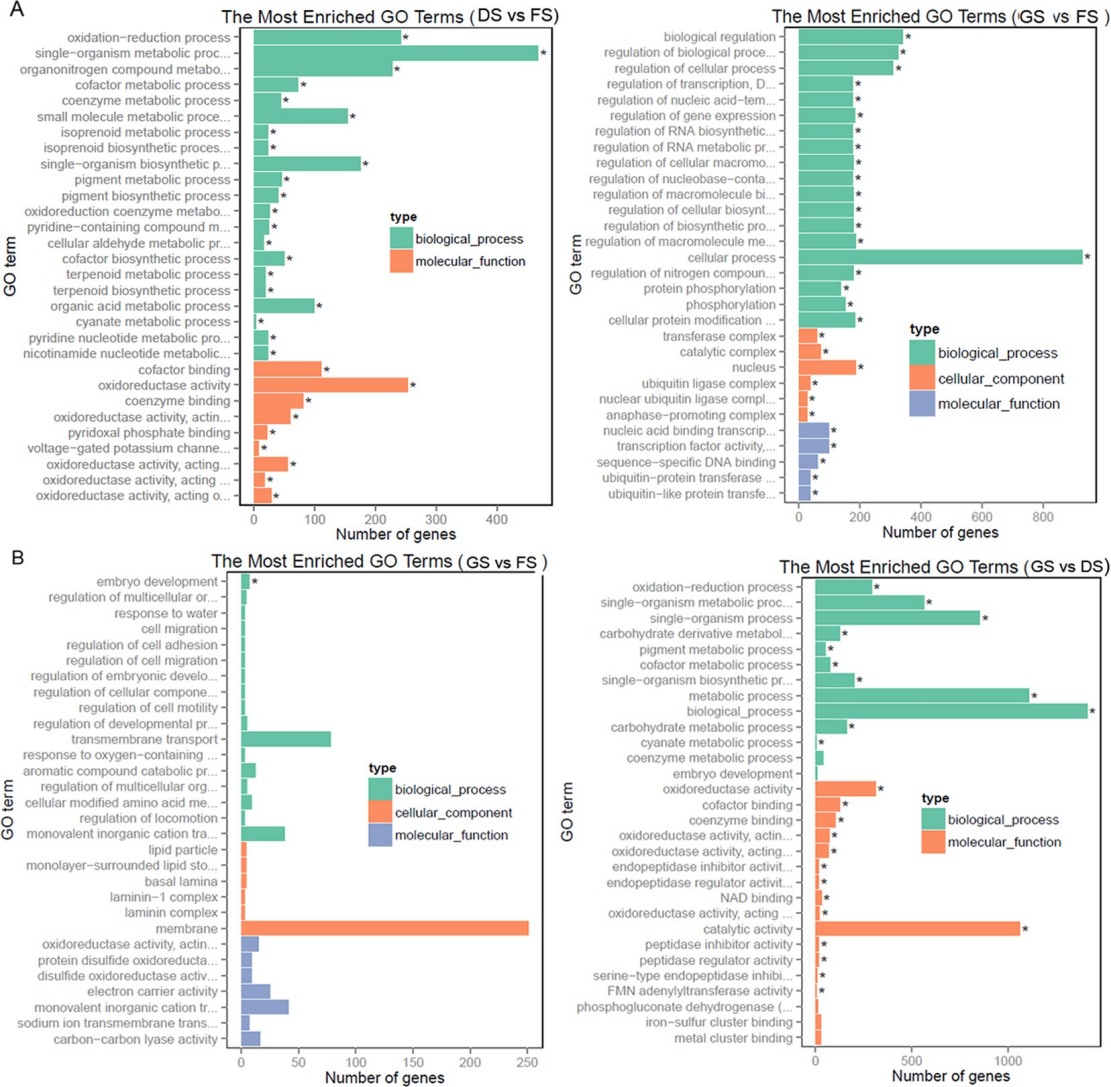
Histogram of DEG functional classification between DS and FS, GS and DS, and GS and FS according to GO annotation. A. Classification of upregulated DEGs; B. Classification of downregulated DEGs.

A GO analysis was performed to explore the biological processes related to dormancy release and germination of peanut kernels. In the DS vs FS comparison, the upregulated DEGs were significantly enriched in 21 GO terms (corrected *p*<0.05), in which the top five terms were oxidation-reduction process (GO:0055114, 241 genes), single-organism biosynthetic process (GO:0044711, 177 genes), organonitrogen compound metabolic process (GO:1901564, 227 genes), cofactor metabolic process (GO:0051186, 78 genes), and small-molecule metabolic process (GO:0044281, 163 genes), while there were no downregulated DEGs markedly enriched in GO terms. In this comparison pair, the majority of the upregulated DEGs were focused on the molecular function of oxidoreductase activity (Fig 3A).When GS and FS were compared, only the downregulated DEGs were grouped to one GO term, that is, the embryo development process (GO:0009790), in which 7 genes out of 20 genes located in the reference genome were detected (Fig 3B). In GS vs DS, the upregulated DEGs were markedly enriched in 19 GO terms, which are involved in several biological regulatory and cellular processes, including regulation of gene expression (GO:0010468, 184 genes), regulation of RNA biosynthetic processes (GO:2001141, 177 genes), protein phosphorylation (GO:0006468, 138 genes), cellular protein modification processes (GO:0006464, 183 genes), and so on; among these processes, the crucial components mainly execute the following molecular functions: nucleic acid-binding transcription factor activity, ubiquitin-protein transferase activity, and so on (Fig 3A). In this pairwise comparison, the downregulated DEGs were mainly grouped to 11 GO terms, among which the majority were detected in the upregulated DEGs in DS vs FS, which included oxidation-reduction processes (GO:0055114, 291 genes), single-organism biosynthetic processes (GO:0044711, 201 genes), cofactor metabolic processes (GO:0051186, 83 genes), etc., and other enrichment terms related to metabolic processes, which mainly included carbohydrate derivative metabolic processes (GO:1901135, 125 genes) and carbohydrate metabolic processes (GO:0005975, 161 genes) (Fig 3B).

### Energy metabolism during after-ripening

A large number of enzymes and mRNAs are involved in the major metabolic pathways, including energy production pathways, and are stored in mature dry seeds in preparation for seed germination and seedling establishment [34–37]. During a period of dry storage, seed dormancy gradually decreases accompanying by the AR process [35]. In our study, the KEGG pathway analysis showed that during the AR of peanut seeds, the expression of many genes involved in oxidative phosphorylation (S2 Table, gmx00190, 48/243 genes, corrected *p* = 2.48e-05), glutathione metabolism (gmx00480, 28/155 genes, corrected *p* = 0.015), and carbon metabolism (S3 Table, gmx01200, 59/447 genes, corrected *p* = 0.021) significantly increased.

**Fig 4:**
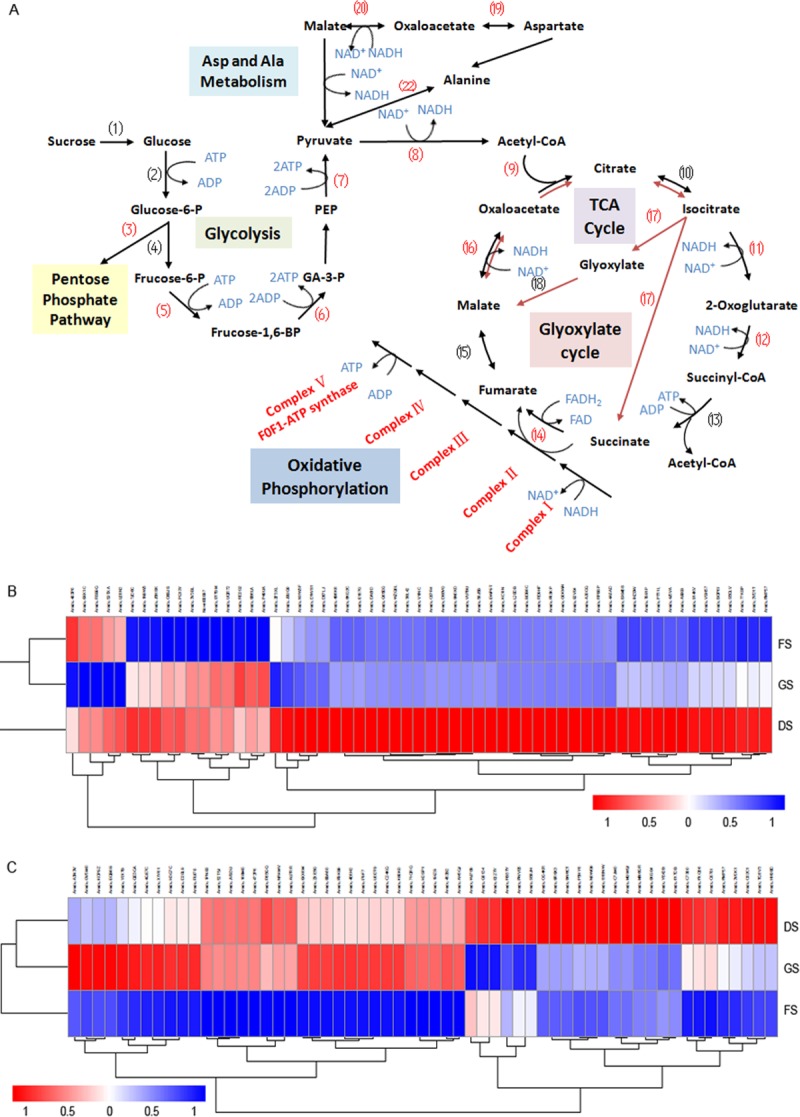
DEGs between the DS and FS stages in several metabolic pathways. A. The majority of upregulated genes represented in the KEGG pathways are involved in glycolysis, the tricarboxylic acid (TCA) cycle, the glyoxylate cycle, ASP and ALA metabolism, and oxidative phosphorylation. (1) Invertase; (2) Hexokinase (HK); (3) Glucose-6-phosphate dehydrogenase (G6PDH); (4) Pyrophosphate-dependent phosphofructokinase (PFP)/diphosphate-fructose-6-phosphate1-phosphotransferase; (5) Phosphofructokinase (PFK); (6) Fructose-biphosphate aldolase (FBA); (7) Pyruvate kinase(PK); (8) Pyruvate dehydrogenase complex; (9) Citrate synthase (CSY); (10) Citrate hydrolyase and citrate hydroxymutase; (11)Isocitrate dehydrogenase (IDH); (12) 2-Oxoglutarate dehydrogenase (OGDH); (13) Succinyl-CoA:acetate CoA transferase/SSA-CoA synthetase; (14) Succinate dehydrogenase (SDH); (15) Fumarate hydratase; (16) Malate dehydrogenase (MDHm); (17) Isocitrate lyase (ICL); (18) Malate synthase (MSY); (19) Aspartate aminotransferase (AspAT); (20) Malate dehydrogenase (MDHc); (21) NAD-dependent malic enzyme 2 (NAD-ME2); (22) Alanine aminotransferase (AlaAT). The numbers in parentheses marked in red represent the upregulated genes encoding the key enzymes in the related pathway. B. Heatmaps of the 59 DEGs among the FS, DS and GS stages involved in the oxidative phosphorylation pathway; C. Heatmaps of the 59 DEGs among the FS, DS and GS stages involved in the carbon metabolic pathway.

In oxidative phosphorylation pathway, a series of protein complexes in the electron transport chain (consisting of complexes I to V) within the inner membrane of mitochondria carried out the sequential redox reactions to oxidize nutrients and to release energy. Our results found that large numbers of the enzyme genes related to this pathway were significantly enriched, which included the genes encoding NADH DEHYDROGENASE (ND) subunits 1, 2, 4, 4L, 5 and 6 as well as NADH DEHYDROGENASE (UBIQUINONE) IRON-SULFUR (NDUFS) subunits1, 2, 7, and 8 and FLAVOPROTEIN 2 (NDUFV2) from complex I (NADH-COENZYME Q OXIDOREDUCTASE); SUCCINATE DEHYDROGENASE (UBIQUINONE) IRON-SULFUR SUBUNIT 2 (SDHB2) from complex II (SUCCINATE-Q OXIDOREDUCTASE); UBIQUINOL-CYTOCHROME C REDUCTASE IRON-SULFUR SUBUNIT (ISP), CYTOCHROME B SUBUNIT (CYTB), CYTOCHROME C1 SUBUNIT (CYT1) and UBIQUINOL-CYTOCHROME C REDUCTASE SUBUNIT 7 (QCR7) from complex III (CYTOCHROME BC1 COMPLEX); CYTOCHROME C OXIDASE (COX) subunits 1, 2 and 3 from complex IV; and different kinds of ATPase subunits from complex V (F-TYPE H^+^-TRANSPORTING ATPASE subunit α; subunits a, b and g; and V-TYPE H^+^-TRANSPORTING ATPASE subunits B, D, E, G, and H as well as a 21 kDa PROTEOLIPID subunit) (Fig 4A and 4B, S2 Table).

In carbon metabolism pathway, many genes associated with glycolysis, the tricarboxylic acid (TCA) cycle (also named the citrate cycle), and the glyoxylate cycle were significantly upregulated during this stage (Fig 4, S3 Table). Among them, twenty-one genes encoding different dehydrogenases, including the PYRUVATE DEHYDROGENASE COMPLEX, ISOCITRATE DEHYDROGENASE (IDH), 2-OXOGLUTARATE DEHYDROGENASE (OGDH), SUCCINATE DEHYDROGENASE (SDH), and MALATE DEHYDROGENASE (MDH), accounted for 1/3 of the upregulated genes, which, by a series of oxidation reactions of intermediates in the glycolysis pathway and TCA cycle, catalyze one pyruvate molecule to produce CO_2_, one molecule of ATP, four NADH molecules and one FADH_2_ molecule [38]. The transcriptional level of four genes encoding aminotransferase (ASPARTATE AMINOTRANSFERASE and ALANINE AMINOTRANSFERASE 2 from mitochondria; SERINE-GLYOXYLATE AMINOTRANSFERASE, and PHOSPHOSERINE AMINOTRANSFERASE 1 from chloroplasts) was markedly elevated in our results (Fig 4A and 4C, S3 Table). The resulting NADH and FADH_2_ molecules enter the electron transport chain and are further oxidized to produce energy by oxidative phosphorylation. In addition, the expression of several genes encoding GLYCERALDEHYDE-3-PHOSPHATE DEHYDROGENASE also increased, which catalyzes the oxidation and phosphorylation of glyceraldehyde-3-phosphate to produce 1,3-bisphospho-D-glycerate in glycolysis.

**Fig 5:**
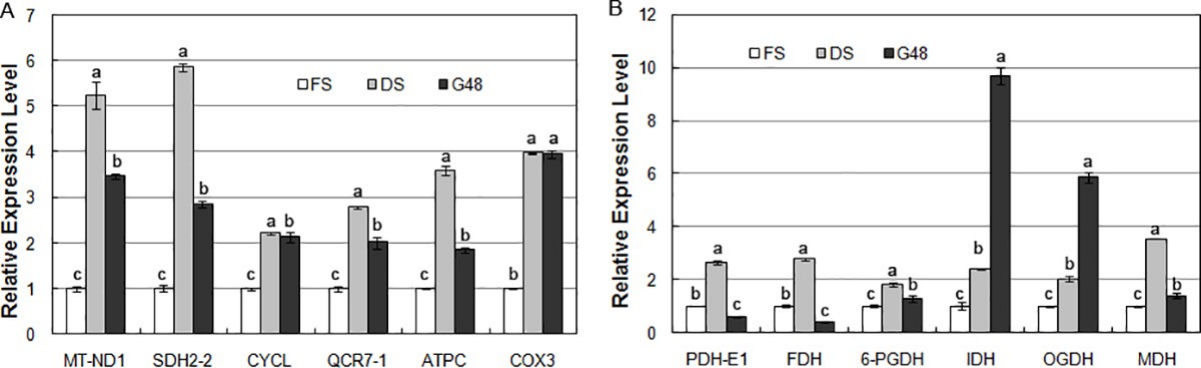
Analysis of the mRNA transcript levels of several DEGs between the DS and FS stages by real-time fluorescent quantitative RT-PCR. A. DEGs related to the electron transport chain in the oxidative phosphorylation pathway; B. DEGs involved in the dehydrogenation reaction in glycolysis and the TCA.

The expression levels of several key genes involved in complexes I to V of the electron transport chain and crucial dehydrogenase genes in the glycolysis pathway and TCA cycle were verified by qRT-PCR. They all displayed the enhanced expression in dry seeds than those in freshly-harvested seeds, while some of them expressed in newly germinated seeds at significantly downregulated way than in dry seeds (Fig 5).

### Multiple pathways of plant hormone signal transduction during seed germination

**Fig 6:**
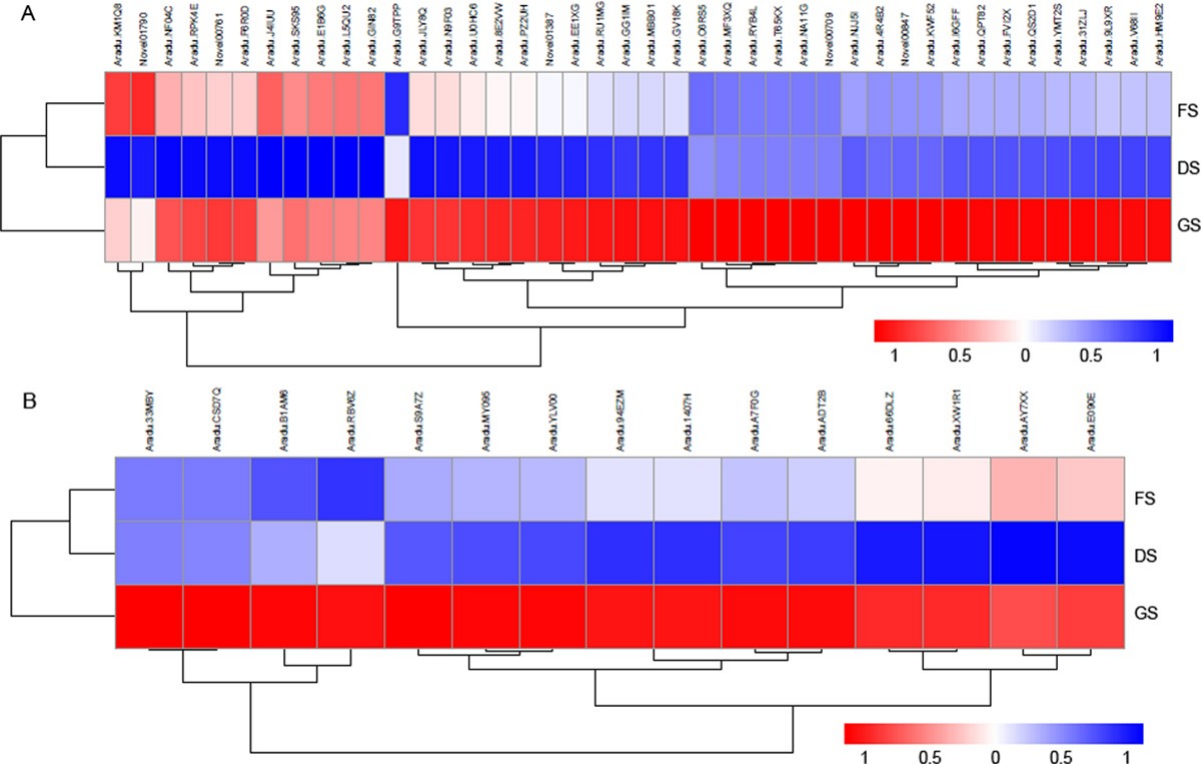
The signaling pathways of phytohormones, including GAs, BRs, auxin, and others, are enhanced in germinated seeds. A. Heatmaps of the 42 DEGs among the FS, DS and GS stages involved in phytohormone signaling pathways; B. Heatmaps of the 15 DEGs among the FS, DS and GS stages involved in the protein ubiquitin degradation pathway.

**Fig 7:**
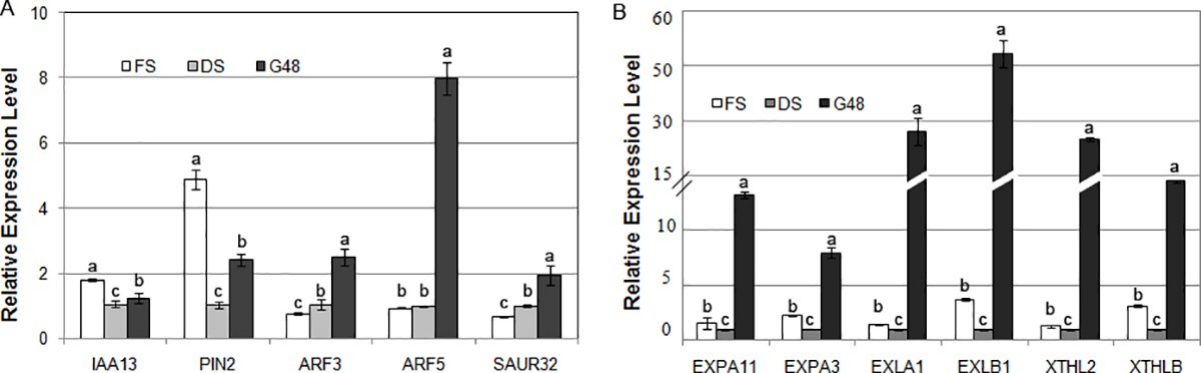
Analysis of mRNA transcript levels of several DEGs between the GS and DS stages by real-time fluorescent quantitative RT-PCR. A. DEGs involved in the auxin signal transduction pathway; B. DEGs related to cell elongation and cell wall remodeling.

During the germination of peanut seeds, some downregulated genes were classified as associated with the oxidative phosphorylation pathway (41 genes, corrected *p* = 0.0025), and dozens of upregulated genes were related to plant hormone biosynthesis and signal transduction (Figs 4B and 6A, S2 and S4 Tables). Indole-3-acetic acid biosynthesis is the necessary trigger for seed germination [6]. The expression of the majority of components in the auxin signal pathway, including gene encoding auxin transporter-like protein AUX1, which is an auxin influx carrier, the F-box protein TRANSPORT INHIBITOR RESPONSE 1 (TIR1) gene, AUXIN RESPONSE FACTOR (ARF) gene, the probable indole-3-acetic acid-amido synthetase GH3 gene, SAUR family protein genes, and AUX/IAA family protein genes, was significantly increased during this period. According to the qRT-PCR results, the expression level of several crucial genes related to IAA signaling was also confirmed to be induced in the germinated seeds (Fig 7A). The expression of many genes involved in brassinosteroid biosynthesis and signal transduction was also markedly increased. These genes included those encoding cytochrome P450, 90B1 and 90A1; the steroid 5α-reductase DET2, the brassinosteroid receptor BRI1 (BRASSINOSTEROID INSENSITIVE 1), BRI1-associated receptor kinase BAK1, and proteins involved in brassinosteroid resistance, BZR1 and BRZ2; however, one *BIN2* gene was downregulated significantly. In addition, the upregulated genes also included some GA and ABA signal transduction genes, for example, the gibberellin receptor gene *GID1*(*GA-INSENSITIVE DWARF 1*), the F-box protein gene *GID2*, the abscisic acid receptor gene *PYR/PYL*, *PROTEIN PHOSPHATASE 2C* (*PP2C*), and the serine/threonine-protein kinase gene *SnRK2* (S4 Table).

Ubiquitin-mediated proteolysis plays an important regulatory role in hormone signaling [39], during which proteins are polyubiquitinated by a reaction cascade that consists of three enzymes named E1 (ubiquitin-activating enzyme), E2 (ubiquitin-conjugating enzyme), and E3 (ubiquitin ligase) that are degraded by the 26S proteasome. Our research showed that the expression of some genes encoding E1, E2, and E3 significantly increased during seed germination, including ubiquitin-like 1-activating enzyme E1 A (UBLE1A), ubiquitin-conjugating enzymes (UBE2C, UBE2D, UBE2I, and UBE2O), CULLIN (CUL1), and the adapter protein SKP1 (S-PHASE KINASE-ASSOCIATED PROTEIN 1), which belong to the SCF complex (multisubunit RING-finger type E3), and so on (S5 Table, Fig 6B).

### Distribution and content of hormones at different seed stages

**Fig 8:**
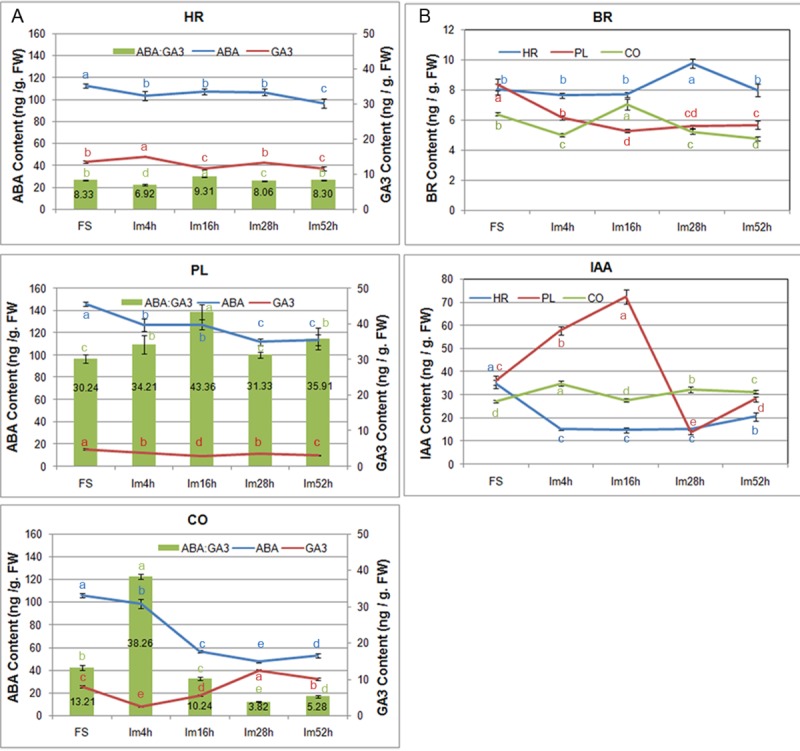
Phytohormone contents in different sections of peanut seeds at different developmental stages. A. ABA and GA contents and the ratio of ABA to GA in freshly harvested seeds, dry seeds, and seeds during imbibition for 4, 16, 28 and 52 h; B. BR and IAA contents in freshly harvested seeds, dry seeds, and seeds during imbibition for 4, 16, 28 and 52 h. HR: hypocotyl and radical; PL: Plumule; CO: Cotyledon.

ABA is a positive regulator of dormancy induction and a negative regulator of germination, while GAs counteract ABA to release dormancy and promote germination. Our detection results showed that ABA contents in every part of the freshly harvested seeds displayed relatively high levels. The ABA contents in COs and PLs rapidly declined during the early phase of seed imbibition and remained at a constant level after imbibition for 28 h, while the ABA content in the HRs slightly increased during the early stage but began to decrease after 28 h. The cotyledons of seeds imbibing for 28 h and 52 h have relatively low ABA levels (47.48 and 52.74 ng/g FW, respectively). GA_3_ levels in the HRs at all development stages, which ranged from 11.51 ng/g FW to 14.94 ng/g FW, were much higher than those in the COs and PLs; GA_3_ levels were lowest in the HRs that imbibed for 16h. In the COs, the GA_3_ content was maintained at a lower level during imbibition for 4 h, after which it increased with the prolongation of imbibition time, peaked during imbibition for 28 h and then slightly decreased. In the PLs, during germination, the GA_3_ content remained at a relatively low level (Fig 8A). Previous studies indicated that it is likely that the ABA/GA ratio, and not the absolute hormone amounts, regulates dormancy release and germination. Dormancy maintenance depends on a relatively high ABA/GA ratio, while dormancy release involves a net shift to increased GA biosynthesis and ABA degradation resulting in a relatively low ABA/GA ratio [7, 11]. Therefore, the ABA/GA_3_ ratios of the three seed parts at all development periods were assessed. The results showed that low ABA/GA_3_ ratios were maintained at all stages in the HRs, while in the PLs, high ABA/GA_3_ ratios occurred at germination period; in the COs, the ABA/GA_3_ ratio of the Im4h seeds was much higher than that at the other stages, and with increased duration of germination, the ABA/GA_3_ ratio sharply decreased from 38.26 to 3.82 (Fig 8A).

BRs and GAs as positive regulators counteract the inhibitory action of ABA and promote cell elongation and seed germination in parallel [11]. Our results showed that in the COs and PLs, BR contents maintained at the lower level and changed slightly during the later stage of seed germination; however, in the HRs, the BR contents were higher than those in other two seed parts at all development stages, and increased significantly after the seeds taking up water for 16h, peaking during imbibition for 28h following a distinct decrease (Fig 8B).

Currently, very little is known about the role of auxin during seed germination. However, some studies found that auxin may interact with GAs, resulting in changes in IAA synthesis and transport during germination [11, 40]. Our results showed that during imbibition for 4 h to 28h, IAA levels were maintained at the lowest levels in the HRs, while they sharply increased and then decreased in the PLs. In addition, soon afterwards the accumulation of IAA increased by small degree in the HRs and PLs with increasing imbibition time. In the COs, IAA levels significantly increased at the early stage of water uptake but then decreased gradually during imbibition for 52 h (Fig 8B).

## Discussion

### The dormancy alleviation of LH14 dry seeds is associated with the rapid resumption of multiple metabolic processes during after-ripening

Seed dormancy is established during seed maturation, and dormancy loss of mature seeds can occur via a period of dry storage (so-called AR), via moist chilling, or via seed coat scarification. Different species or the same species living in various natural habitats have evolved to have different dormancy adaptions to different environmental conditions. For instance, the Landsberg *erecta* (Ler) and Columbia (Col) ecotypes of *Arabidopsis* have a low level of dormancy, while the seeds of the Cape Verde Islands (Cvi) ecotype show strong dormancy [10]. AR is a specific developmental procedure that broadens or increases the sensitivity of the perception of seeds to environmental conditions for promoting germination while simultaneously decreasing or narrowing the sensitivity of perception in repressing germination [7, 41]. The duration of the AR process varies greatly among species: some last for several days, and others last for several months or more [10, 15, 25].The physiological status of dry seeds seems to be quiescent; however, abundant changes in gene expression have been found in dry seeds compared to dormant seeds, which are triggered by AR [42–44]. It was shown that some genes associated with storage mobilization and cell wall modification were more highly expressed in AR seeds compared with dormant seeds of *Arabidopsis* and wheat [42, 43]. Although transcriptional profiles and molecular mechanisms underlying dormancy release by AR are conserved between species, there are unique regulatory mechanisms in different species [43, 45]. In this study, LH14 seeds displayed an obvious AR period during dry storage despite easily losing their dormancy. The results of the transcriptomic analysis of the dormant and AR peanut seeds indicated that many genes (3440 genes) were differentially expressed, of which the majority were involved in multiple metabolic processes, including oxidative phosphorylation, carbohydrate metabolism, and glutathione metabolism. After-ripened seeds with dormancy have performed the necessary preparations for germination and seedling establishment, in which the mobilization of reserves and energy production play crucial roles. In dry peanut seeds, we found that large amounts of key genes associated with glycolysis, the TCA and glyoxylate cycles, and amino acid metabolism were highly transcribed. It was suggested that stored soluble carbohydrates, fatty acids, amino acids and other intermediates could be rapidly utilized by the resumption of several metabolic pathways and that early activation of oxidative phosphorylation by the electron transport chain could produce large amounts of ATP to supply the following procedure. Some studies in soybean, *Arabidopsis* and sugar beet indicated that glycolysis, fermentation, the TCA and glyoxylate cycles and the oxidative pentose phosphate pathway (OPPP) are quickly activated in AR seeds following imbibition to supply energy for germination [15, 26, 34, 37]. Therefore, like in dry peanut seeds, several catalytic procedures involved in energy metabolism might be rapidly resumed during AR of dry seeds in other dicotyledonous species; only dry peanut seeds with nondeep dormancy are highly sensitive to AR. The key genes corresponding to these pathways may still be expressed at a relatively high level during the early phase of peanut seed germination, while the majority displays downregulated expression patterns during the late period of germination and testa breakage.

### The coordination of hormone signal transduction nets plays a key role in radicle protrusion

During the period of radicle protrusion, the breakage of the seed testa and the elongation of hypocotyl are the major visible characteristics. Various phytohormones and environmental signals take part in the regulation of these processes.

The antagonistic effects of GAs and ABA on seed dormancy breakage and germination have been clarified in many monocot and dicot species [1, 7, 11, 46]. During the processes of seed maturation, induction and maintenance of dormancy, dormancy release, and seed germination, the contents of ABA and GAs in seeds and the sensitivity of seeds to those hormones exhibit complex dynamic relationships. Although GA accumulation correlates with dormancy release and germination, GA treatment alone apparently does not satisfy the conditions of seed germination. A reduction in ABA levels is a prerequisite before GA contents and sensitivity begin to increase [47]. Our results found that the amount of ABA in the cotyledons sharply decreased at the onset of seed imbibition and was maintained a relatively constant level during the late phase of imbibition, while the GA_3_ level continued to increase after sowing during imbibition for 4h and remained steady after imbibition for 28 h. Clearly, prior to the increase in GA_3_ content, the ABA content decreased to a low level, and ABA/GA_3_ ratio dropped down correspondingly. In fact, maintaining dormancy requires a relatively high ABA/GA ratio, while dormancy breakage and germination depend on the complementation of increasing GA biosynthesis and ABA degradation, resulting in a relatively low ABA/GA ratio [7, 11, 47]. And, GA is helpful for increasing the growth potential of embryo and weakening the tissue covering the radicles [11]. Thus, the resulting relatively low ABA/GA_3_ ratio in peanut cotyledons is beneficial for radicle protrusion through the testa. However, we did not find any genes involved in GA synthesis whose expression markedly increased in germinated seeds, but we found that four significantly upregulated genes participated in GA signal transduction, two of which encode the GA receptor GID1 and two of which encode the F-box protein GID2, which is a major component of the SCF^SLY1/GID2^ ubiquitin E3 ligase complex. In addition, during this period, the expression of a *DELLA* gene (gmx: 547719), which represses the GA signal transduction cascade, was significantly downregulated. In the GA signaling module, GA and the receptor GID1 together with DELLA repressors form the GA-GID1-DELLA complex. When the bioactive GA level increases, GID1 combined with GA undergoes a conformational change, and DELLA is recruited to the SCF^SLY1/GID2^ ubiquitin E3 ligase complex for polyubiquitination and subsequent degradation by the 26S proteasome, relieving the suppressive effects on downstream GA-responsive genes [20, 48]. We also found that some genes involved in the ubiquitin-mediated proteolysis pathway are markedly upregulated in germinated seeds, including one gene encoding a SCF ubiquitin ligase complex protein. It is implied that the sensitivity of GA perception may increase during initial radicle emergence, while the increasing of GA content in the cotyledon was attributed to the GA transport from the place of biosynthesis. Furthermore, GAs trigger seed germination by removing the mechanical restraint of the seed coat and endosperm, during which the expression of some *EXPANSIN* (*EXP*) and *XYLOGLUCAN ENDOTRANSGLYCOSYLASE* (*XET/XTH*) family members is induced [37, 49–52]. Our results indicated that the expression levels of eight peanut *EXP* genes [including five *α-EXPANSIN* (*EXPA*), one *EXPANSIN-LIKEA* (*EXPLA*), two *EXPANSIN-LIKE B* (*EXPLB*)] and six *XTH* genes]were significantly upregulated at the seed germination stage compared to those at the fresh or dry seed stage (Fig 7B).

Both GAs and BRs stimulate seed germination by different regulatory mechanisms [47, 53, 54]. Although both GAs and BRs can induce the expression of cell elongation- and cell wall organization-related genes, including *EXP*s, these hormones promote the expression of distinct family members. BRs are considered to promote seed germination by directly improving the growth potential of embryos in a GA-independent manner [47, 54]. When the BR content is high, BRs are perceived and bound by BRI1, and they activate the BRI1/BAK1 kinase complex, which therefore inhibits the downstream repressors of BR signaling, including the GSK3-like kinase BIN2; the inhibition of BIN2 results in the accumulation of unphosphorylated BZR1/2 family transcription factors that regulate BR-target gene expression [12, 55–57]. In the present study, the expression levels of some transcripts associated with positively regulating BR biosynthesis and the BR signaling pathway were markedly upregulated during the germination of peanut seeds, including *DET2*, *BRI1*, *BAK1*, *BRZ1*, and so on, while expression of the *BIN2* gene (gmx:100802451), which encodes a kinase that negatively regulates BR signal transduction, was markedly downregulated. Moreover, we found that the significant increase in BR levels occurred only in the hypocotyls and radicles of imbibed peanut seeds. It is suggested that the elevation of BR content in the HR section of the imbibed peanut embryos was rapidly perceived by BRI1 and that the BR signal transduction cascade was subsequently initiated, resulting in some genes required for cell elongation being activated in the imbibed hypocotyls and radicles until seed germination finished. This idea is consistent with the conclusion that an increase in BR signaling intensity improves the status of seed germination and increases the length of hypocotyls [56, 58].

Auxin is a major hormone associated with plant morphogenesis and is also essential for promoting hypocotyl elongation and seed germination [58–60]. The SCF^TIR1^–auxin–AUX/IAA complex is the central component of the auxin signaling model; which auxin triggers the ubiquitination and degradation of AUX/IAA family proteins to derepress the inhibition of ARF transcription factors, subsequently promoting the expression of some auxin-responsive genes [61]. In this study, at the radicle protrusion time point, the expression of a large number of genes involved in auxin signal transduction (*AUX1*, *TIR1*, *CULLIN*, *AUX/IAAs*, *ARFs*, *GH3*, *SAUR*) substantially increased; the transcription levels of two *PIN2* genes (encoding an auxin efflux carrier) also significantly increased. At this time, no obvious expression changes were detected for genes specifically involved in auxin biosynthesis. Taken together, these results imply that during the late stage of peanut seed germination, auxin transport is active and that the auxin signaling pathway plays important regulatory roles. Auxin distribution in young *Arabidopsis* seedlings is imbalanced, with relatively high levels in the root apex and cotyledons, and its polar transport is associated with root morphogenesis and gravitropism [62, 63]. We found that during the early stage of imbibition, the IAA contents in the CO and PL sections of peanut seeds rapidly increased to a relatively high level, especially in the PL section, while in the HR section, it remained constant until germination approached, after which it started to rise slowly; these findings indicated that the modest increase in IAA levels may be related to the elongation of the embryo axis (HR section) during germination.

BR- and auxin-mediated cell elongation is interdependent, and this synergism does not depend on the level of hormone biosynthesis [64]. The crosstalk between auxin and BR signals is found to converge on the regulation of ARF transcription factors, which act downstream from BZR1 and AUX/IAA proteins and trigger the expression of some auxin-response genes with the ARFAT motif (TATCTC) in their promoter [49, 64]. Walcher and Nemhauser (2012) found that BZR2 and ARF5 could bind to the 5′ flanking region of the *SAUR15* gene that is activated by both auxin and BRs [65]. Oh et al. recently clarified a central regulatory module in regulating hypocotyl cell elongation in which auxin, BRs, GAs, light, and temperature signals were integrated together. In this module, BZR1 and the light-responsive factor PIF4 coregulate hypocotyl cell elongation by interacting with specific ARFs, such as ARF6 and ARF8. However, the GA signal pathway in regulating cell elongation converges by removing the inhibitory effects of DELLA proteins on specific ARFs together with BZR1 and PIF4 to promote the expression of some ARF target genes [58]. At the germination time point of peanut seeds, the homologues of *Arabidopsis* genes *ARF3*, *ARF5*, *ARF8*, *ARF18* and *ARF32* were significantly upregulated. However, it is unclear which among these genes is the central one or the one that integrates the auxin signal with BR and GA signals. This question needs to be tested by experiments. Several studies have indicated that the seed germination process is involved in the regulation of cell expansion and cell wall organization, during which the expression levels of some *XTH* and *EXP* genes improve markedly [37, 49–52, 59, 65]. Recent studies have shown that the 5′ flanking region of some *XTH*, *EXP* and other auxin-responsive genes, including *IAA*s, *SAUR*s and *GH3*s, contain a TGTCTC motif or its inverse element GAGACA, which is the binding site of some ARFs [58, 59, 64, 65]. Our results also showed that many of the genes mentioned above were expressed at a high level in the newly germinated seeds, suggesting that some specific ARFs together with other transcription factors that cooperatively regulate GAs, BRs, auxin, and other signals modulate ARF target gene expression and promote the breakage of peanut seed testa and hypocotyl elongation.

## Supporting information

S1 Table The statistical analysis on the germination rates of peanut seeds. (DOCX)

S2 Table DEGs involved in the oxidative phosphorylation pathway. (XLSX)

S3 Table DEGs involved in the carbon metabolism pathway. (XLSX)

S4 Table DEGs involved in the phytohormone signaling pathways. (XLSX)

S5 Table DEGs involved in the ubiquitin mediated proteolysis pathway. (XLSX)
