# Supplementary material for: Transcriptional Differences in Peanut (Arachis hypogaea L.) Seeds at the Freshly Harvested, After-ripening and Newly Germinated Seed Stages: Insights into the Regulatory Networks of Seed Dormancy Release and Germination
Source: PLoS One. 2020 Jan 3;15(1):e0219413. doi: 10.1371/journal.pone.0219413 (PMC6941926; doi:10.1371/journal.pone.0219413)
Supplement: S1 Table — (DOCX) [file pone.0219413.s001.docx]

**S1 Table. The statistical analysis on the germination rates of peanut seeds**

| **Accessions** | **Status (Fr/Dr)** | **Days of Germination** | | | | | | | |  |
| --- | --- | --- | --- | --- | --- | --- | --- | --- | --- | --- |
|  |  | **3d** | | **4d** | | **5d** | | **6d** | | |
| LH14 | Fr | 0.0±0.0a | C | 2.8±0.0a | C | 44.4±2.8a | D | 58.3±2.8a | C | |
|  | Dr | 91.7±7.3b |  | 100±0.0b |  | 100±0.0b |  | 100±0.0b |  |  |
| FH1 | Fr | 2.8±0.0a_1_ | D | 2.8±0.0a_1_ | D | 52.8±4.8a_1_ | D | 66.7±7.4a_1_ | C | |
|  | Dr | 58.3±7.4b_1_ |  | 86.1±4.8b_1_ |  | 88.9±2.8b_1_ |  | 97.2±4.8b_1_ |  |  |
| LGMK | Fr | 0.0±0.0a_2_ | C | 2.8±0.0a_2_ | CD | 72.2±7.4a_2_ | C | 77.8±5.6a_2_ | B | |
|  | Dr | 94.4±2.8b_2_ |  | 94.4±2.8b_2_ |  | 97.2±2.8b_2_ |  | 97.2±2.8b_2_ |  |  |
| CHI | Fr | 47.2±7.4a_3_ | B | 77.8±10.0a_3_ | B | 86.1±9.6a_3_ | B | 100±0.0a_3_ | A | |
|  | Dr | 83.3±7.4b_3_ |  | 94.4±4.8b_3_ |  | 100±0.0b_3_ |  | 100±0.0a_3_ |  |  |
| SLH | Fr | 50.0±7.35a_4_ | A | 88.9±5.6a_4_ | A | 100±0.0a_4_ | A | 100±0.0a_4_ | A | |
|  | Dr | 97.2±4.8b4 |  | 100±0.0b_4_ |  | 100±0.0a_4_ |  | 100±0.0a_4_ |  |  |
| **Factor** | | ***F*** | | ***F*** | | ***F*** | | ***F*** | | |
| **Accessions** | | 58.0** | | 177.3** | | 50.6** | | 47.2** | | |
| **Status** | | 1053.0** | | 1458.0** | | 265.1** | | 204.2** | | |
| **Accessions ×Status** | | 35.1** | | 145.8** | | 35.0** | | 41.5** | | |

One-way analyses of variance (ANOVA) were used to analyze the effects between two different status (fresh or dry seeds) of one variety on germination ratio, and two-ways ANOVA were applied to estimate the effects among different varieties on germination ratio. Mean differences were determined based on the least significant difference (LSD) at the P＜0.05 probability level, and shown alphabetically in order a, b,…, and so on. The significant differences in *F* value of different factors at 1% level were shown as double asterisk “**”.
